# A Randomised Controlled Trial of a Brief Online Mindfulness-Based Intervention in a Non-clinical Population: Replication and Extension

**DOI:** 10.1007/s12671-017-0856-1

**Published:** 2018-01-16

**Authors:** Kate Cavanagh, Alasdair Churchard, Puffin O’Hanlon, Thomas Mundy, Phoebe Votolato, Fergal Jones, Jenny Gu, Clara Strauss

**Affiliations:** 1 School of Psychology, University of Sussex, Falmer, East Sussex BN1 9QH UK; 2 Sussex Partnership NHS Foundation Trust, Hove, BN3 7HZ UK; 3 School of Psychology, Politics and Sociology, Canterbury Christ Church University, Kent, UK

**Keywords:** Mindfulness, Meditation, Mediation, E-mental health, Internet intervention, Self-help

## Abstract

Building on previous research, this study compared the effects of two brief, online mindfulness-based interventions (MBIs; with and without formal meditation practice) and a no intervention control group in a non-clinical sample. One hundred and fifty-five university staff and students were randomly allocated to a 2-week, self-guided, online MBI with or without mindfulness meditation practice, or a wait list control. Measures of mindfulness, perceived stress, perseverative thinking and anxiety/depression symptoms within were administered before and after the intervention period. Intention to treat analysis identified significant differences between groups on change over time for all measured outcomes. Participation in the MBIs was associated with significant improvements in all measured domains (all *p*s < 0.05), with effect sizes in the small to medium range (0.25 to 0.37, 95% CIs 0.11 to 0.56). No significant changes on these measures were found for the control group. Change in perseverative thinking was found to mediate the relationship between condition and improvement on perceived stress and anxiety/depression symptom outcomes. Contrary to our hypotheses, no differences between the intervention conditions were found. Limitations of the study included reliance on self-report data, a relatively high attrition rate and absence of a longer-term follow-up. This study provides evidence in support of the feasibility and effectiveness of brief, self-guided MBIs in a non-clinical population and suggests that reduced perseverative thinking may be a mechanism of change. Our findings provide preliminary evidence for the effectiveness of a mindfulness psychoeducation condition, without an invitation to formal mindfulness meditation practice. Further research is needed to confirm and better understand these results and to test the potential of such interventions.

## Introduction

Mindfulness has been described as “the awareness that emerges through paying attention on purpose, in the present moment, and non-judgmentally to the unfolding of experience moment by moment” (Kabat-Zinn 2003, p. 145). Mindfulness-based interventions (MBIs) teach individuals to observe, acknowledge, accept and decentre from thoughts, feelings and emotions that come into awareness, and do not aim to change direct experience (thoughts, feelings, bodily sensations, etc.), but rather encourage a changed way of relating to it (Kabat-Zinn et al. 1985; Shapiro et al. 2006). There is growing evidence that MBIs can have positive consequences for psychological (Brown and Ryan 2003; Keng et al. 2011) and physical (Grossman et al. 2004) health, in both clinical (Chiesa and Serretti 2011; Hofmann et al. 2010; Strauss et al. 2014; Vøllestad et al. 2012) and non-clinical (Eberth and Sedlmeier 2012; Khoury et al. 2015) populations.

Given the measured benefits of mindfulness, the possibility of extending the reach of MBIs through lower-intensity interventions has recently begun to be explored. The most widely used protocols, mindfulness-based stress reduction (MBSR) and mindfulness-based cognitive therapy (MBCT), involve approximately 26 to 30 group therapy hours (an initial orientation session, 8 × 2 or 2.5 h sessions and a whole day workshop), coupled with an invitation to engage in daily home practice during the 8-week intervention period. However, recent research findings suggest that alternative methods of offering MBIs, including web-based interventions (Spijkerman et al. 2016) and self-help approaches (Cavanagh et al. 2014) may also hold promise.

Mindfulness-based self-help books, computer programmes, smart phone apps, audio and video recordings of mindfulness exercises and especially guided mindfulness-orientated meditations are widely available in the public domain. There is growing evidence that use of these kinds of interventions may be associated with increases in mindfulness and reductions in depression and anxiety in comparison to control conditions (Cavanagh et al. 2014). For example, MBIs by self-practice with phone guidance in clinical populations (Niles et al. 2013; Thompson et al. 2010), and in non-clinical populations through self-practice guided by CD (Warnecke et al. 2011), or book and CD (Lever Taylor et al. 2014) have proven beneficial. The popularity of mindfulness-based interventions in non-clinical populations may reflect a desire for people to develop strategies to cope with the stresses and strains of daily life and to build resilience against developing mental and physical health difficulties in the future.

There are also a large number of mindfulness resources available online. A recent survey indicated that many people prefer online formals for mindfulness meditation interventions above group or individual face-to-face approaches (Wahbeh et al. (2014)), and Segal (2011) has noted that the evolution of MBIs is likely to be in the delivery of online programmes. The majority of these resources offer information about mindfulness and/or audio recordings of guided mindfulness meditation (e.g. www.mindful.org/resources). Guided 8-week online mindfulness programmes have demonstrated comparable efficacy in comparison to active control conditions (Ly et al. 2014; Thompson et al. 2010), and unguided 8-week online mindfulness programmes have demonstrated efficacy in comparison to wait list conditions in randomised controlled trials (Boettcher et al. 2014; Morledge et al. 2013).

The preliminary evaluation of briefer online interventions has also indicated some positive results, with one group reporting evidence of reductions in perceived stress (Krusche et al. 2012) and symptoms of anxiety and depression (Krusche et al. 2013) in open trials of a 4-week programme, and another indicating that mindfulness can be enhanced through a 2-week unguided self-help intervention and that use of the programme was also associated with reductions in perceived stress and improvements in self-reported symptoms of anxiety and depression in a university sample (Cavanagh et al. 2013). However, it should be noted that not all studies of briefer online MBIs report unequivocally positive results. Glück and Maercker (2011) found that whilst their 2-week web-based, self-guided, mindfulness training was acceptable and subjectively beneficial to many users, intent-to-treat analysis found no difference between the mindfulness intervention group and a wait list control on measures of mindfulness, global distress, perceived stress or mood post-training or at 3 months follow-up.

Overall, this emerging research points to the potential of online mindfulness-based self-help interventions and growth in this area and the development and evaluation of further online mindfulness training programmes has been anticipated (cf. Monshat et al. 2012). In addition to the potential for rapid and widespread dissemination of MBIs offered by the use of online resources, the computerisation of such interventions lends itself to component analyses and dismantling research which may help to address a number of pressing questions about their active ingredients (Marks and Cavanagh 2009; Cavanagh et al. 2014).

Both ancient and contemporary writings in the Buddhist tradition (Olendzki 2009), and MBI manuals (e.g. Kabat-Zinn 1990; Segal et al. 2013), propose that experiential learning through the regular practice of formal mindfulness meditation is essential for the cultivation of mindfulness. Both MBCT and MBSR emphasise the importance of formal mindfulness meditation. These involve being guided by the facilitator through meditation practices of between 3 and 40 min in which participants are invited to bring mindful awareness to various aspects of their experience (the breath, body, movement, sounds and thoughts). Approximately half of the group therapy time is devoted to these mindfulness practices, and daily home meditation practices of 30–40 min, supported through audio recordings, are an integral part of both programmes.

Regular mindfulness meditation is thought to be necessary for improved psychological well-being, since it is the ability to be mindful in the moment that is believed to facilitate symptom reduction and protection against relapse, and it is suggested that this ability can only be cultivated through regular practice. However, there is a lack of consensus in the broader literature about whether duration, frequency or type of meditation (formal or informal) is most important (Crane et al. 2014) and few studies have tested such claims empirically. Some findings show an association between extent of mindfulness practice, increases in mindfulness and symptom reduction (Carmody and Baer 2008; Shapiro et al. 2003) and risk of relapse (Crane et al. 2014) in MBIs, whilst others have not (Carmody and Baer 2009; Hindman et al. 2015; Shapiro et al. 2007). This inconsistent pattern of findings may partly reflect the challenge of reliably measuring the amount and quality of mindfulness practice that people engage with (Del Re et al. 2013). In a naturalistic study of MBIs, Hawley et al. (2014) found that engaging in formal, planned mindfulness meditations, but not informal mindfulness practices (such as using breathing spaces in response to stress, mindful eating) was associated with decreased rumination and symptom alleviation. Data from Williams et al.’s (2014a) randomised controlled dismantling study suggests that formal mindfulness practice may be an active ingredient of MBCT for a subgroup of people with a history of recurrent depression and suicidality who have experienced greater severity of childhood trauma. Thus, it appears that, for at least some MBIs and participant groups, formal practice may play an important role. Where it does apply, we should see added value for MBIs that include formal mindfulness practice in comparison to the same intervention excluding formal practice.

The second aim of this study is to investigate the potential of brief MBIs to impact perseverative thinking patterns. Perseverative thinking patterns such as rumination and worry are key components of depression and anxiety, and MBIs may be useful in the treatment or reduction of these unhelpful thinking styles (Querstret and Cropley 2013). There is evidence that the effects of mindfulness training on reduction in mental health symptoms are mediated by reductions in rumination and worry (Gu et al. 2015). Indeed it is suggested that mindfulness reduces mood symptoms in depression only to the extent that it reduces these mediating cognitive processes. It may be that individuals who practice mindfulness on a regular basis become more able to engage in effective attention regulation strategies when they experience dysphoric affect (Hawley et al. 2014).

There is some evidence that brief (4 week) group mindfulness meditation training is associated with reductions in rumination in comparison to a control group (Jain et al. 2007), but to date, studies of briefer online MBIs have not considered the effects of these interventions on perseverative thinking. This study measures changes in perseverative thinking in the context of a brief online mindfulness intervention. Furthermore, our component analysis design allows us to test the proposal that experiential learning through formal mindfulness meditation practice is necessary for this process of change (Segal et al. 2013).

In summary, the present study is a replication and extension of a previous study (Cavanagh et al. 2013), which found that, compared to a wait list control group, a brief online MBI had positive consequences for mental health symptoms in a university sample. The present study replicates the two arms of this previous study (formal mindfulness meditation condition and wait list condition) and measures mindfulness, anxiety/depression and perceived stress outcomes in a university sample. The present study also extends this previous study by adding a mindfulness psychoeducation only arm and a perseverative thinking outcome measure—a proposed mechanism of effective MBIs. This study aims to contribute to the MBI literature (i) by testing the hypothesis that formal mindfulness meditation is an active ingredient of brief MBIs by using a component analysis design and (ii) by investigating the effects of these brief interventions on perseverative thinking—a proposed mechanism of effective MBIs. Better understanding of the active ingredients and mechanisms of online MBIs should support the development of more effective and efficient versions of these interventions.

## Method

### Participants

Inclusion criteria were that participants were students or staff at the host university, and this was ensured as the intervention was hosted on the university’s secure virtual learning system which was only accessible to students and staff. No exclusion criteria were applied. Participants were 155 (124 female) students (*n* = 120) and staff (*n* = 35) from a university in the South of England who had responded to either a recruitment email or posters that had been placed around the university campus. Age ranged from 18 to 68 years (*M* = 31.03 years, SD = 11.64 years). Whilst no inclusion or exclusion criteria based on participant’s distress was employed during this study, it is of note that 56% of our sample scored at least one standard deviation over the general population mean on the PHQ-4 measure (cf. Löwe et al. 2010), and 81% scored at least half a standard deviation above a community mean on the perceived stress scale (Cohen and Williamson 1988; cf. Rose et al. 2013). Moreover, more than half of the sample (61%) scored at or above the cut-off for likely caseness of anxiety (31%), depression (6%) or both (24%; Kroenke et al. 2009). The study protocol was approved by the ethics committee at the host university and informed consent was obtained from each participant prior to participation.

### Procedure

The study was advertised on the university campus. All participants signed up to the ‘Learning Mindfulness Online’ course, gave informed consent and then completed the baseline questionnaires online, hosted on the Bristol Online Survey platform. A number of studies have now demonstrated that paper-and-pencil and Internet data collection methods are generally equivalent, including measures of perceived stress and symptoms of depression (e.g. Herrero and Meneses 2006). Within 24 h of completing the baseline questionnaires, participants were randomised, by a researcher blind to participant details, using a computer-generated blocked random allocation method (block size 15), to either start the mindfulness psychoeducation intervention immediately (mindfulness psychoeducation condition), to start the mindfulness meditation intervention immediately (mindfulness meditation condition) or to join a wait list control condition. Participants were blind to hypotheses regarding the role of formal mindfulness meditation in this study, and were informed that they would be assigned to “a version of the ‘Learning Mindfulness Online’” or a waiting list condition.

The intervention groups were given instant access to their allocated version of the ‘Learning Mindfulness Online’ course and were encouraged to log-on to become familiar with their site. Those assigned to the waiting list condition were informed that they would be invited to join the ‘Learning Mindfulness Online’ course 1 month later.

#### The Online Mindfulness-Based Interventions

The ‘Learning Mindfulness Online’ interventions were delivered using the university’s virtual learning facility, built with an open source learning management system, Moodle. Materials included a streaming video, embedded text and an article to be downloaded and read with a pdf reader. This virtual learning facility is accessible via a university login on any web-enabled device on or off campus. Both interventions included identical information about mindfulness, advice on applying the principles of mindfulness to activities of daily living (informal practice), an invitation to record mindful activities, a journal to reflect on experiences of mindfulness, study information, help and assistance (study information sheet, contact email for researchers, university counselling services and mental health charities).

Information about mindfulness was presented in a brief video (5 min), embedded text (900 words) and a downloadable pdf (2000 words) each of which describe mindfulness as paying attention in the present moment, with openness and curiosity, instead of judgement. This included the idea of bringing attention to our bodily sensations, thoughts and feelings as well as to present moment external events. Guidance on applying mindfulness to daily life is also included (850 words); this recommends choosing one routine activity per day in week 1 (brushing your teeth, drinking tea, coffee or juice, loading the washing machine, etc.) and offers a daily guided walking exercise for week 2 of the intervention. The ‘formal practice’ intervention also included information about mindfulness meditation practice and audio practices that users were invited to follow daily. Participants were given access to the intervention for a period of 14 days; all elements of their assigned interventions were available from the beginning. The researcher teams’ email address provided was for technical difficulties only, beyond that the programme was self-guided, without personal contact.

In the formal practice intervention, participants were invited to listen daily to a 10-min audio track that contained a guided, mindfulness sitting meditation. The meditation practice was adapted from Person-Based Cognitive Therapy (Chadwick 2006) and MBCT (Segal et al. 2013) and is one that has been used in three previous published studies (Chadwick et al. 2016; Dannahy et al. 2011; Strauss et al. 2012). The mindfulness practice was associated with significant improvements in self-reported mindfulness in an RCT for intervention participants relative to those in the control condition (Strauss et al. 2012), and a qualitative study including this mindfulness practice generated themes similar to themes reported in MBIs with more prolonged mindfulness practices (Strauss et al. 2015). Two versions of the10-min meditation were provided, so the participants could choose to listen to a female or male voice, as they preferred. Both versions were recorded by experienced clinical psychologists who were also accredited MBCT practitioners. The audio recording invites participants to adopt a comfortable, upright sitting position and guides participants to bring non-judgemental attention first to the body (from the feet to the head), then the breath and finally to thoughts and feelings.

Participants in both conditions also received standardised reminder emails at 3-day intervals, with four reminder emails being sent in total. Each reminder email invited participants to continue with the intervention, and contained ‘hints and tips’ for their mindfulness practice. In week one, these consisted of general mindfulness practice information, e.g. ‘there is no right or wrong way to practice mindfulness’. In the second week, they provided suggestions on ways in which mindfulness could be brought into participants’ everyday life, e.g. mindful eating, mindful walking.

The standardised emails were sent every 3 to 4 days and, after 2 weeks, all participants received a standardised email with a direct link to the end of study questionnaire. Participants received three reminder emails in total for the closing questionnaire. Those who completed the closing questionnaire received a final email thanking them for their participation. All waiting list participants were then enrolled onto the ‘Learning Mindfulness Online’ course and given access to the full learning mindfulness online programme.

### Measures

#### Five Facet Mindfulness Questionnaire (FFMQ; Baer et al. 2006)

This 39-item self-report scale is used to measure changes in participant’s tendency to be mindful in daily life. Participants are asked to what extent each of the statements are true of them. Each item is on a five-point Likert-type scale from 1—*never or very rarely true* to 5—*very often or always true*. We report on a composite measure including four of the original five FFMQ subscales (excluding ‘observe’). Psychometric papers on the FFMQ have supported the use of a total scale score (omitting scores from the observe subscale) in addition to subscale scores (e.g., Baer et al. 2008; Gu et al. 2016; Williams et al. 2014b).The FFMQ scale reported showed good internal consistency at baseline in this sample, Cronbach’s alpha = 0.93.

#### Perceived Stress Scale (PSS; Cohen and Williamson 1988)

The 10-item Perceived Stress Scale (PSS) is designed to measure how unpredictable, overloaded or uncontrollable participants have found their lives. The scale asks participants to rate how often they have felt or thought they had been out of control, overloaded and unpredictable during the last 2 weeks on a five-point Likert-type scale from 0—*never* to 4—*very often*. The PSS showed good internal consistency at baseline in this sample, Cronbach’s alpha = 0.89.

#### Patient Health Questionnaire for Depression and Anxiety (PHQ-4)

The PHQ-4 is a brief screening measure for anxiety and depression, focusing on experiences during the previous 2 weeks (Kroenke et al. 2009). Four items are answered on a four-point Likert scale ranging from 0—*not at all*, to 3—*nearly every day*, an example item being: “Over the past two weeks have you been feeling down, depressed, or hopeless?” Total score is determined by adding together the scores for each of the four items. Scores are rated as normal (0–2), mild (3–5), moderate (6–8) and severe (9–12). The PHQ-4 showed good internal consistency at baseline in this sample, Cronbach’s alpha = 0.84.

#### Perseverative Thinking Questionnaire (PTQ; Ehring et al. 2011)

The PTQ measures dysfunctional repetitive negative thinking as a transdiagnostic process, independent of disorder-specific thought content. Respondents characterise their typical responses to negative experiences by rating 15 statements, such as “I feel driven to continue dwelling on the same issue” on a five-point Likert scale (0—never to 4—almost always). Confirmatory factor analysis suggests a single higher-order factor and three lower-order factors (Ehring et al. 2011). Only the total score was used in our analysis. This decision was supported by exploratory factor and correlational analyses conducted on the baseline scores, which unambiguously yielded a single factor and high correlations between the proposed subscales (Pearson’s *r* 0.76 to 0.82). The PTQ showed good internal consistency at baseline in this sample Cronbach’s alpha = 0.96.

#### Engagement and Experience Questionnaire

At baseline, participants indicated their previous meditation experience using a five-point Likert scale (“How much experience of meditation do you have?”, 1 = none to 5 = 5+ years). After the 2-week intervention period, they were asked to indicate how often they had practiced mindfulness meditation (“How often have you practiced mindfulness meditation over the last two weeks?”) and how often they had applied the principles of mindfulness to activities of daily living during the 2-week intervention, (“How often have you applied the principles of mindfulness to your activities during the last two weeks?” 1 = not at all to 5 = at least once a day), how frequently they intended to continue to practice (“How frequently do you intend to continue practicing mindfulness?”, 1 = not at all to 5 = at least once a day) and how frequently they had read intervention related emails (“Did you read the reminder emails from the mindfulness research group?”, 1 = never to 5 = always). In order to assess participants’ experience of the mindfulness online intervention, they were asked how beneficial they thought the 2-week intervention had been for them (“Do you think practicing mindfulness during the past 2 weeks was beneficial for you?”, 1 = not at all to 5 = very beneficial).

### Data Analyses

For tests of intervention effectiveness, missing data were replaced using the baseline-observation carried forward (BOCF) method (Gupta 2011). These analyses therefore included all participants who entered the study irrespective of completion of post-intervention questionnaires. BOCF makes the assumption that intervention participants who fail to complete post-intervention measures did not benefit and therefore is a conservative method of taking account of missing data. This method is therefore likely to underestimate the true effect of an intervention (Liu-Seifert et al. 2010). Completer analyses are also reported.

To determine the effects of intervention condition on mindfulness, stress, perseverative thinking and symptoms, the primary analyses performed using IBM SPSS version 24 were 3-way (group) analyses of covariance (ANCOVA) on pre-post change in FFMQ, PSS, PHQ-4 and PTQ scores, including baseline measures of PHQ-4 and PTQ as covariates to control for baseline differences between groups on these measures. Significant main effects were explored using Helmert contrasts to compare the intervention groups to each other, and the mean of both intervention conditions to the wait list control. Simple contrasts (to look at change over time within each condition), their effect sizes (*d*) and 95% confidence intervals (CIs) were calculated using Eqs. 4, 15 and 18 in Nakagawa and Cuthill (2007).

**Fig. 1:**
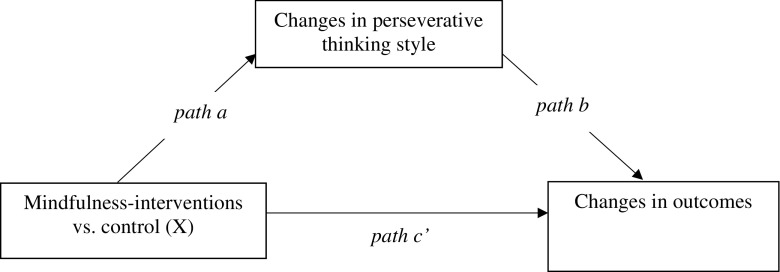
Path diagram depicting the stage two mediational model, with changes in perseverative thinking style as the mediator

As recommended (Kazdin 2007), only complete data sets were used for the mediation analyses. Mediation analysis was conducted on completer sample data to test the hypothesis that improvements in perseverative thinking would mediate the relationship between condition and improvements in stress, anxiety and depression. Bootstrapped 95% bias-corrected confidence intervals were calculated with 5000 resamples using MPlus (demo version 7.2), a syntax-driven programme for estimating a wide range of structural equation models, including mediation models (Muthén and Muthén 1998–2012). This approach was used instead of the causal steps approach (Baron and Kenny 1986) as it is both more powerful and more robust to violations of assumptions of multiple regression analysis (Preacher and Hayes 2008). This provides the 95% confidence intervals of the indirect effect of the intervention group on improvements in stress and anxiety/depression through the proposed mediator (improvements in perseverative thinking). This effect is deemed significant if the 95% confidence intervals do not cross zero. Non-bootstrapped path coefficients were also calculated for the effect of the intervention group on the mediator (path a), the effect of the mediator on the dependent variable whilst controlling for the independent variable (path b) and the effect of the intervention group on the dependent variable whilst controlling for the mediator (path c’; see Fig. 1). As the independent variable, condition, is multicategorical but not dichotomous (with three levels: mindfulness meditation, mindfulness psychoeducation and wait list control), we used Hayes and Preacher’s (2014) MPlus code to implement their recommended method of mediation analysis with a multicategorical independent variable. This estimated a single model for each dependent variable (perceived stress and anxiety/depression symptom severity). In both models, the mindfulness meditation condition was used as the reference category; the contrasts were mindfulness meditation versus mindfulness psychoeducation, and mindfulness meditation versus wait list control.

## Results

One hundred and fifty-five participants were randomly allocated to either the mindfulness psychoeducation intervention, the mindfulness meditation intervention or to the waiting list condition. At baseline, 25 participants (16%) reported that they currently practiced mindfulness, with differences between groups just failing to reach statistical significance (see Table 1). People who had a current mindfulness practice at baseline scored significantly higher on the FFMQ, *t*(153) = 2.80, *p* = 0.006, and significantly lower on the PSS, *t*(153) = −2.21, *p* = .029, than non-meditators. Baseline differences between mindfulness meditators and non-meditators on the PHQ4 and PTQ were not significant, *t*(153) = −1.24, *p* = 0.22 and *t*(153) = − 0.82, *p* = 0.41, respectively). Characteristics of the samples are provided in Table 1.

**Table 1:** Characteristics of the mindfulness and wait list control groups at baseline (*n* = 155)

Variable	Mindfulness psychoeducation intervention		Mindfulness meditation intervention		Wait list control		Statistics
	Mean	SD	Mean	SD	Mean	SD	*F*(2, 152) = 0.9, *p* = 0.92
Age (years)	30.92	11.78	31.54	11.45	30.60	11.90	
Gender	N	%	N	%	N	%	*X*^2^(2) = 1.0, *p* = 0.59
Male	12	23%	8	15%	11	22%	
Female	41	73%	44	85%	39	78%	
Practice mindfulness at baseline	12	23%	10	19%	3	6%	*X*^2^(2) = 5.94, *p* = 0.051

Of the 155 participants randomised in this study, 105 (68%) completed questionnaires at pre- and post-intervention. There was no significant difference in completion rate across groups (*X*^2^(2, *n* = 155) = 4.62, *p* = 0.06), although a greater percentage of complete data sets were available for the wait list group (80%) than the mindfulness-intervention groups (mindfulness psychoeducation = 65%, mindfulness meditation = 58%) completing the post-intervention questionnaires. No significant differences between study completers and those who dropped out were found with respect to age or baseline scores on the measures of mindfulness, stress, perseverative thinking and depression/anxiety (*p*s > 0.1). Males were significantly more likely to contribute a full set of data to the study than females (*X*^2^(1, *n* = 155) = 8.49, *p* = 0.01).

**Fig. 2:**
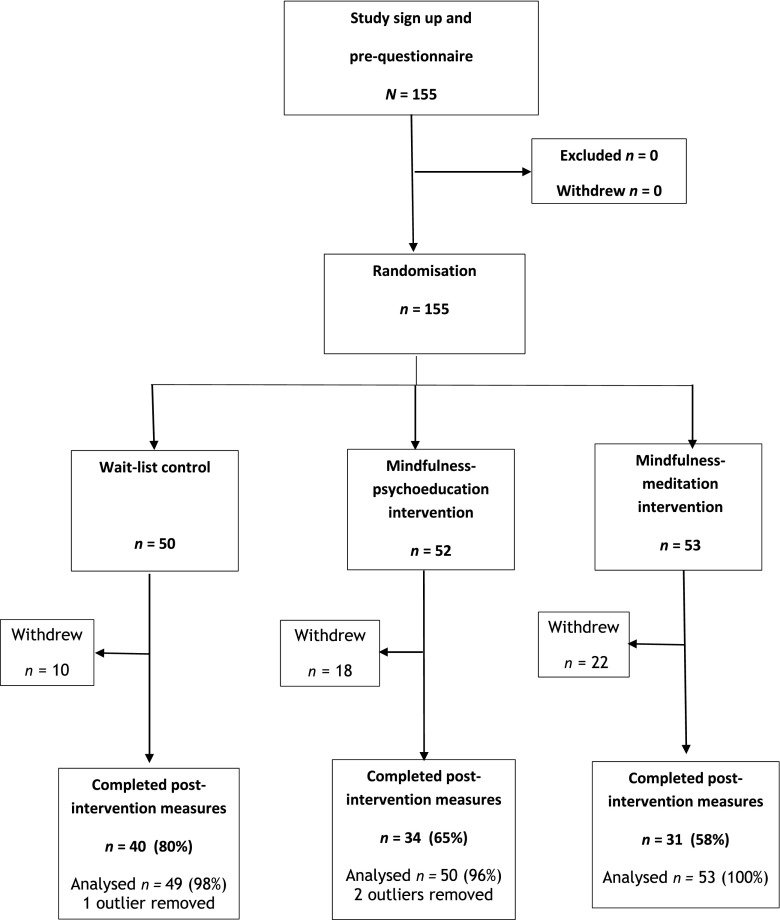
CONSORT diagram outlining the process of service user flow through the study

Preliminary data screening found three outliers (1 wait list, 2 mindfulness psychoeducation) who scored more than 2.5 standard deviations from the mean FFMQ total score at baseline. Data for these participants was removed from subsequent analysis to avoid them having a disproportionate influence on the ANOVA models. Data for all participants were available at baseline (Table 1), and all remaining participants (*n* = 152) were included in analyses using the BOCF method to replace missing data (see Fig. 2).

Pre- and post-scores on the outcome measures are shown in Table 2, with the BOCF method used to replace missing post-intervention data. Significant between-group differences were found on baseline measures of symptoms of anxiety/depression (PHQ-4) and perseverative thinking (PTQ), and therefore, analyses of covariance (ANCOVA) are used to test for between-group differences controlling for baseline scores on these measures. We tested the homogeneity of regression slopes assumption for each outcome variable and the independence of covariate and treatment effect assumption and both assumptions were met, indicating that ANCOVA was appropriate for the data.

**Table 2:** Descriptive and inferential statistics comparing the mindfulness interventions to the wait list control group on mindfulness, perceived stress and anxiety/depression symptoms (*n* = 152)

Variable	Mindfulness psychoeducation intervention		Mindfulness meditation intervention		Wait list control		*F*(2, 147)
	Pre	Post	Pre	Post	Pre	Post	
FFMQ	114.52 (21.55)	123.00^a^ (19.12)	111.36 (22.06)	118.57^a^ (21.63)	108.71 (16.75)	110.80^b^ (16.45)	3.35, *p* = 0.01
PSS	21.58 (7.13)	18.92^b^ (7.53)	23.23 (6.46)	20.85^b^ (6.47)	24.18 (6.43)	23.76^a^ (7.05)	3.56, *p* = 0.03
PHQ-4	4.46 (2.96)	3.52^b^ (3.00)	5.98 (3.18)	4.91^b^ (3.36)	5.51 (3.35)	5.65^a^ (3.47)	6.42, *p* < 0.01
PTQ	33.12 (13.42)	30.42^b^ (12.66)	38.87 (11.26)	35.36^b^ (12.92)	36.49 (10.67)	36.27^a^ (10.59)	3.27, *p* = 0.04

Standard deviations are shown in parentheses. *F* is for ANCOVAs performed on change scores controlling for baseline PHQ-4 and PTQ scores; planned contrasts revealed significant differences (*p* < 0.05) between groups; this is marked in superscript, a > b

*FFMQ* Five Facet Mindfulness Questionnaire, *PSS* Perceived Stress Scale, *PHQ-4* Patient Health Questionnaire, *PTQ* Perseverative Thinking Questionnaire

### Mindfulness

ANCOVA showed a significant main effect of group on pre-post intervention change scores in mindfulness as measured by FFMQ controlling for baseline PHQ-4 and PTQ (*F*(2147) = 4.08, *p* = 0.02). Helmert contrasts showed significantly greater improvement in mindfulness in the intervention conditions in comparison to the wait list control (contrast estimate = − 4.35, *p* < 0.01) but no significant difference between intervention groups (contrast estimate = 2.04, *p* = 0.28). Simple contrasts showed that whilst scores for the waiting list group remained unchanged from pre- to post-intervention (*t*(48) = 1.47, *p* = 0.15, *d* = 0.09, 95% CI for *d* − 0.03 to 0.21), there was a significant increase over time in mindfulness in both the mindfulness psychoeducation intervention group (*t*(49) = 4.05, *p* < 0.001, *d* = 0.35, 95% CI for *d* 0.17 to 0.53) and the mindfulness meditation intervention group (*t*(52) = 4.38, *p* < 0.001, *d* = 0.25, 95% CI for *d* 0.11 to 0.40)*.*

### Perceived Stress

ANCOVA showed a significant main effect of group on pre- to post-intervention perceived stress change scores measured by total PSS scores, controlling for baseline PHQ-4 and PTQ (*F*(2147) = 3.56, *p* = 0.03). Helmert contrasts showed significantly greater pre-post improvement in perceived stress in the intervention conditions in comparison to the wait list control (contrast estimate = −2.12, *p* = 0.01), but no significant difference between intervention groups (contrast estimate = 0.51, *p* = 0.59). Simple contrasts showed that whilst scores for the waiting list group remained unchanged from pre- to post-intervention (*t*(48) = 0.61, *p* = 0.55, *d* = 0.06, 95% CI for *d* − 0.14 to 0.27), a significant decrease in perceived stress scores was found both in the mindfulness psychoeducation intervention group (*t*(49) = 4.22, *p* < 0.001, *d =* 0.37, 95% CI for *d* 0.18 to 0.55 and the mindfulness meditation intervention group (*t*(52) = 3.84, *p* < 0.001, *d* = 0.37, 95% CI for *d* 0.17 to 0.56).

### Anxiety and Depression

ANCOVA showed a significant main effect of group on pre-post anxiety and depression change scores as measured by total PHQ-4 scores and controlling for baseline PHQ and PTQ (*F*(2, 147) = 6.42, *p* < 0.01). Helmert contrasts showed significantly greater pre-post improvement in anxiety/depression in the intervention conditions in comparison to the wait list control (contrast estimate = − 1.20, *p* < 0.01), but no significant difference between intervention groups (contrast estimate = − 0.9, *p* = 0.82). Simple contrasts showed that whilst scores for the waiting list group remained unchanged from pre- to post-intervention (*t*(48) = 0.44, *p* = 0.66, *d* = 0.04, 95% CI for *d* −0.14 to 0.23), a significant decrease in anxiety and depression was found both in the mindfulness psychoeducation intervention group (*t*(49) = 3.84, *p* < 0.001, *d =* 0.32, 95% CI for *d* 0.14 to 0.49) and the mindfulness meditation intervention group (*t*(52) = 4.14, *p* < 0.001, *d* = 0.33, 95% CI for *d* 0.16 to 0.50.

### Perseverative Thinking

ANCOVA showed a significant effect of group on pre- to post-intervention perceived stress change scores measured by total PSS scores, controlling for baseline PHQ-4 and PTQ scores (*F*(2, 147) = 3.27, *p* = 0.04). Helmert contrasts showed significantly decreased perseverative thinking from pre- to post-intervention in both intervention conditions in comparison to the wait list control (contrast estimate = 2.80, *p* = 0.012), but no significant difference between intervention groups (contrast estimate = 0.39, *p* = 0.76). Simple contrasts showed that whilst scores for the waiting list group remained unchanged from pre- to post-intervention (*t*(49) = 0.28, *p* = 0.78, *d* = 0.02, 95% CI for *d* − 0.13 to 0.17), a significant pre-post decrease in perseverative thinking scores was found in both the mindfulness psychoeducation intervention group (*t*(49) = 2.41, *p* = 0.02, *d* = 0.36, 95% CI for *d* 0.18 to 0.54) and the mindfulness meditation intervention group (*t*(52) = 3.94, *p* < 0.001, *d* = 0.28, 95% CI for *d* 0.13 to 0.42).

### Completer Analysis

All of the main analyses reported above were also conducted on a completer sample only data set (*n* = 105), and the statistical significance of effects remained unchanged in all cases. Within-group effects for the wait list control condition remained negligible and non-significant for all outcomes. For the mindfulness psychoeducation and mindfulness meditation conditions, within-group pre-post changes increased towards the moderate range of magnitude for all outcomes (range *d* = 0.38–0.69, compared to *d* = 0.25–0.37 for BOCF).

### Mediation Analyses

**Fig. 3:**
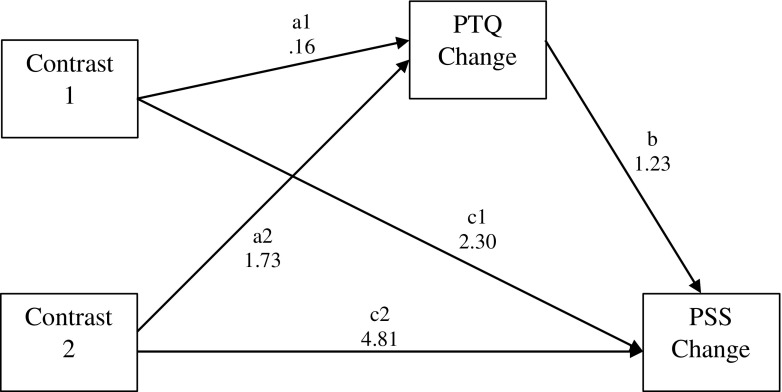
Path diagram depicting model 1, testing whether improvements in perseverative thinking (PTQ Change) mediate the effects of mindfulness meditation versus psychoeducation (Contrast 1) or mindfulness meditation versus wait list control (Contrast 2) on improvements in perceived stress (PSS Change). Unstandardised path coefficients are displayed

**Fig. 4:**
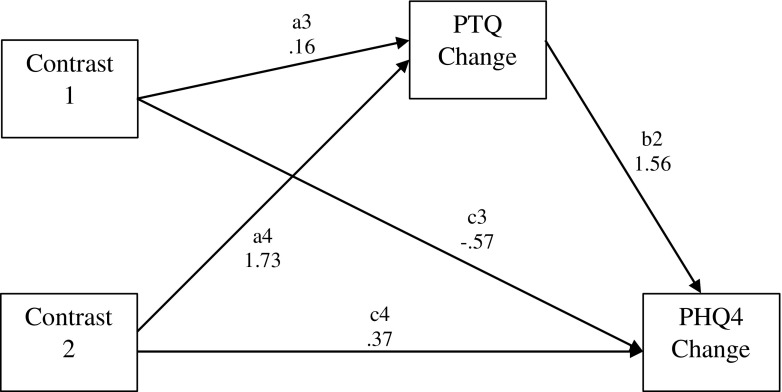
Path diagram depicting model 2, testing whether improvements in perseverative thinking (PTQ Change) mediate the effects of mindfulness meditation versus psychoeducation (Contrast 1) or mindfulness meditation versus wait list control (Contrast 2) on improvements in anxiety/depression symptom severity (PHQ4 Change). Unstandardised path coefficients are displayed

Table 3 reports the results of the MPlus analysis for the two multicategorical independent variable mediation models that tested whether perseverative thinking mediates the relationship between condition and improvement in perceived stress and anxiety/depression symptom severity for the wait list control group and mindfulness psychoeducation condition in comparison to the mindfulness meditation condition. The first model included perceived stress as the outcome and the second model included anxiety/depression symptom severity as the outcome. Figures 3 and 4 present path diagrams of these models.

**Table 3:** Unstandardised regression coefficients, their standard errors (SEs) and significance values, and bootstrapped 95% bias-corrected confidence intervals for the two multicategorical independent variable mediation models with PTQ as the proposed mediator

	*B*	SE	*t*	*p*	95% BC CIs^a^
Model 1. With perceived stress as the dependent variable					
Contrast 1: mindfulness meditation vs. mindfulness psychoeducation					-1.22 to 1.62
*a* path: group ⇨ PTQ change	0.16	0.54	0.30	0.76	
*b* path: PTQ change ⇨ PSS change	1.23	0.37	3.38	0.001	
*c’* path: group ⇨ PSS change (direct effect)	2.30	2.22	1.04	0.30	
Indirect effect (a × b)	0.20	0.70	0.29	0.77	
Contrast 2: mindfulness meditation vs. wait list control					0.89 to 4.26*
*a* path: group ⇨ PTQ change	1.73	0.58	3.00	0.003	
*b* path: PTQ change ⇨ PSS change	1.23	0.37	3.38	0.001	
*c’* path: group ⇨ PSS change (direct effect)	4.81	1.65	2.92	0.003	
Indirect effect (a × b)	2.14	0.83	2.58	0.01	
Model 2: With anxiety/depression as the dependent variable					
Contrast 1: mindfulness meditation vs. mindfulness psychoeducation					-1.45 to 1.85
*a* path: group ⇨ PTQ change	0.16	0.54	0.30	0.76	
*b* path: PTQ change ⇨ PHQ4 change	1.56	0.19	8.18	< 0.001	
*c’* path: group ⇨ PHQ4 change (direct effect)	− 0.57	1.10	− 0.52	0.60	
Indirect effect (a × b)	0.26	0.85	0.30	0.76	
Contrast 2: mindfulness meditation vs. wait list control					0.96 to 4.62*
*a* path: group- > PTQ change	1.73	0.58	3.00	0.003	
*b* path: PTQ change- > PHQ4 change	1.56	0.19	8.18	< 0.001	
*c’* path: group - > PHQ4 change (direct effect)	0.37	1.00	0.37	0.71	
Indirect effect (a × b)	2.71	0.94	2.90	0.004	

*BC CIs* bias-corrected confidence intervals, *PHQ4* Patient Health Questionnaire (anxiety and depression), *PSS* Perceived Stress Scale, *PTQ* Perseverative Thinking Questionnaire

^a^Bootstrapped 95% BC CIs for the a × b effect; a significant indirect effect is indicated by * where these do not cross zero (*p* < 0.05)

Perseverative thinking was found to mediate the relationship between participation in the mindfulness meditation versus the wait list control condition and improvements in perceived stress and anxiety/depression symptom severity, as the bootstrapped 95% confidence intervals for the a × b effect do not cross zero in any case. No mediation effects were found comparing mindfulness psychoeducation and mindfulness meditation intervention conditions on stress or anxiety/depression symptom severity as the bootstrapped 95% confidence intervals for the a × b effect cross zero in all cases.

### Engagement and Experience

Among those who completed the post-test measures, almost everyone in both intervention groups reported applying mindfulness principles to daily life (mindfulness psychoeducation condition = 31/34 = 91%, mindfulness meditation condition = 29/31 = 94%) and engaging in mindfulness practice during the 2-week intervention period (mindfulness psychoeducation condition = 29/32 = 91%, mindfulness meditation condition = 29/31 = 94%), with differences between groups being non-significant (respectively, ***χ***^**2**^(65) = 0.13, *p* = 0.72 and ***χ***^**2**^(63) = 0.38, *p* = 0.54). The frequency on a 1–5 scale (1 = none at all, 5 = at least once a day) of reported mindfulness practice was also not different between the two intervention groups (mindfulness psychoeducation median = 4.00, 25% = 3.00, 75% = 5.00), mindfulness meditation median = 4.00, 25% = 4.00, 75% = 4.00; *U* = 438.50, *p* = 0.53). In contrast, only a minority of the wait list participants reported applying mindfulness principles to daily life (5/39 = 13%) or practicing mindfulness (5/40 = 13%).

Most people in the intervention groups reported reading the reminder emails and finding them useful at least sometimes (mindfulness psychoeducation = 26/34 = 76%, mindfulness meditation = 20/31 = 65%). They also perceived some benefit from their engagement with the programme (mindfulness psychoeducation = 28/34 = 82%, mindfulness meditation = 26/31 = 76%), and planned to continue to practice mindfulness in the future (mindfulness psychoeducation = 28/34 = 82%, mindfulness meditation = 26/31 = 76%). No significant differences were found between the intervention groups for how much they thought they had learnt about mindfulness (*t*(53) = 2.43, *p* = 0.218), their perceived benefit (*t*(53) = 1.13, *p* = 0.794) or intended future practice frequency (*t*(53) = 0.62, *p* = 0.137).

## Discussion

Our RCT compared two brief online mindfulness-based self-help interventions (with and without a mindfulness meditation component) to a waiting list control group in a large unselected university population. The results suggest that these brief interventions, offered with or without an invitation to formal daily mindfulness meditation practice, can increase mindfulness and reduce perseverative thinking, perceived stress and symptoms of anxiety and depression, with small to medium magnitudes of effect in intention to treat analysis. These findings replicate and extend previous findings reported by our group (Cavanagh et al. 2013).

The brief MBIs were found to be associated with significant reductions in perseverative thinking, shown to be a key mechanism of action for the effects of traditional MBIs on symptom outcomes (Gu et al. 2015). Mediation analyses found that change in perseverative thinking mediated the relationship between condition and improvement on both perceived stress and anxiety/depression symptom severity outcomes for the mindfulness meditation condition in comparison to the wait list control condition, but not when the mindfulness meditation condition was compared to mindfulness psychoeducation. This suggests that the mindfulness meditation intervention may work in the same way as longer interventions, by reducing perseverative thinking.

No significant differences were found in the effects of the MBIs with or without a mindfulness meditation component, and the study had a sufficient sample size to detect a medium-sized difference between the two mindfulness groups (i.e. *d* = 0.56, *f* = 0.28) with a power set at the conventional level of 0.8. One interpretation of these findings is that formal mindfulness meditation practices are not necessary to gain the potential benefits available from brief mindfulness interventions in non-clinical populations, and that practicing mindfulness informally in daily life in the absence of formal meditation practice may be sufficient to lead to measurable change. This interpretation runs contrary to the hypothesis that regular formal practices are essential in order to build mindfulness potential (Hawley et al. 2014; Segal et al. 2013). However, an alternative interpretation may be found in the self-reported practice data. We found no difference in self-reported engagement with the principles and practice of mindfulness, including mindfulness practice between the two intervention conditions. This may reflect a misunderstanding on the part of the mindfulness psychoeducation group leading to over reporting of mindfulness practice, or perhaps the invitation to mindfulness through a mindfulness psychoeducation intervention encouraged participants in our university sample to seek out their own mindfulness meditation practices (which are widely available online and on campus), which could account for the similarity on reporting of engagement and similarity in measured outcomes between our mindfulness intervention conditions. Future research using diary methods could illuminate this finding. A third possibility is that, in the context of self-help interventions containing brief mindfulness practices, the distinction between formal and informal mindfulness practice may be more apparent than real. After all, perhaps we might expect similar change in levels of mindfulness (and other associated outcomes) if participants pay non-judgmental attention to their breath and body during a short sitting practice or if they bring the same quality of attention, for a similar amount of time, to the act of cleaning their teeth, for example. There is some evidence that brief mindfulness practices (10 min) in face-to-face mindfulness-based interventions for people experiencing depression can confer similar benefits to learning mindfulness than the longer practices (30–40 min) included in MBCT (Strauss et al. 2015). Nonetheless, it may be the case that the longer formal mindfulness practices found in MBSR and MBCT might show additional benefits in comparison to informal mindfulness practice, as it seems likely that it would be more challenging to maintain informal mindfulness practice for 30 min than to follow a 30-min, formal, CD-guided practice. It would be interesting for future research to test this possibility.

Participants completing the MBIs typically reported high levels of engagement with the intervention, including regular practice and reading the intervention emails. Most of those accessing the brief mindfulness interventions stated that they had learnt about mindfulness from the interventions, that the interventions had been of benefit to them and that they intended to continue to practice mindfulness in the future. This supports the idea that brief online mindfulness interventions may be a meaningful route into mindfulness practice for some people. Future research should aim to identify for whom such interventions are likely to be most attractive and most beneficial.

This extends a small, but promising, body of research suggesting that brief, self-guided, online, MBIs may offer benefits for non-clinical groups seeking stress-reduction strategies and indicating the potential of online (Spijkerman et al. 2016) and self-help (Cavanagh et al. 2014) methods for increased dissemination of MBIs. These findings also complement a growing body of evidence supporting the potential benefits of the internet as a means to extend the reach of psychological therapies (e.g. Cuijpers et al. 2015; Grist and Cavanagh 2013; Marks and Cavanagh 2009) and other public health interventions (e.g. Bennett and Glasgow 2009; Gulliver et al. 2015).

The effect sizes reported are in the small-medium range and, where measured, similar in magnitude to those reported in a previous trial of the same intervention (Cavanagh et al. 2013) and from recent meta-analyses of the effects of other mindfulness and acceptance-based self-help interventions (Cavanagh et al. 2014; Spijkerman et al. 2016). The magnitude of effects of these brief interventions on self-reported mindfulness fall below those reported in the meta-analysis of studies of group mindfulness-based interventions reported by Visted et al. (2015, pre-post effect for mindfulness groups 0.53, 95% confidence interval (CI) = 0.46–0.61). Effects on anxiety/depression symptoms are also smaller than those reported in a pre-post meta-analysis of studies of more intense mindfulness based in interventions reported by Hofmann et al. (Hofmann et al. 2010, pre-post effects for mindfulness-based interventions 0.63 (95% CI = 0.53, 0.73) for reducing anxiety and 0.59 (95% CI = 0.51, 0.66) for reducing depression, although both of these meta-analyses included studies of participants with elevated symptoms at baseline which may account for some of the magnitude of change found in their analyses. Future research should explore the clinical significance of brief mindfulness intervention effects to determine if such interventions are likely to be of real benefit to public health.

Whilst attrition from studies of conventional mindfulness and acceptance-based interventions are typically quite low (mean 15%, range 0–44%, Vøllestad et al. 2012), attrition from mindfulness-based online (range 8–60%; Spijkerman et al. 2016) and self-help (mean = 32%, range 7–50%; Cavanagh et al. 2014) interventions may be somewhat higher. The attrition rates in this study reflect this, with approximately six out of ten participants in the interventions conditions completing post-intervention measures. This summary data and those from the current study are more in line with the relatively high attrition rates found for other self-guided internet interventions (Christensen et al. 2009; Waller and Gilbody 2009). Completer analysis indicated a larger magnitude of effects for those participants completing both pre- and post-intervention measures. How online content is presented and delivered can have an impact on appeal and adherence and may be improved through the development of more technically advanced digital packages. Objective data on engagement with the online programme was not collected during this study, and so it is unclear whether self-reported data on intervention engagement accurately reflects this. Future research should include collection of objective engagement data, follow-up data on reasons for drop-out and intervention development should explore methods by which to maximise engagement and minimise counter-therapeutic attrition from mindfulness-based self-help interventions.

Therapist-supported self-help interventions are typically associated with better outcomes and higher rates of adherence in comparison to pure self-help (Gellatly et al. 2007; Richards and Richardson 2012; Spijkerman et al. 2016), and the potential added value of brief support for MBIs online should also be explored. These interventions were brief, offered limited content and limited interactivity. Further methods of reducing attrition via manipulation and optimisation of both the programme content and the user experience should also be explored and evaluated (cf. Barazzone et al. 2012; Cavanagh and Millings 2013).

### Limitations

Study attrition was quite high, but no significant differences between study completers and those who dropped out were measured in age, gender or baseline mindfulness, stress or anxiety/depression. To compensate for this high rate of attrition, all data-analyses were conducted on intention to treat data, using the conservative BOCF imputation method. This method is likely to underestimate the true effect of the intervention (Liu-Seifert et al. 2010). Completer analyses are also reported.

The effect of both interventions could be attributable to non-specific effects of being actively involved in an intervention programme (emails, website guidance, etc.), which participants in the wait list control condition did not receive. Furthermore, participants may have expected benefits of taking part in a mindfulness intervention and improvements in outcomes could be wholly or partially accounted for by these expectations of benefit and/or demand characteristics (i.e. participants in the active intervention arms reporting improvement at post-intervention in line with researcher expectations), rather than the intervention itself. Future research employing a well-matched non-mindfulness-based control group is needed to test this possibility.

The study was not designed to test a non-inferiority hypothesis, indeed our expectation was that the mindfulness psychoeducation condition would function as a placebo control. Therefore, further adequately powered research is needed to test the non-inferiority hypothesis that emerges from this preliminary study finding no measurable difference between intervention condition with and without formal mindfulness medication practice.

The study recruited an unselected university sample, who were 80% of whom were female, and so any extrapolation of the findings to stressed and distressed community samples, or clinical groups should be made with caution. However, our sample reported relatively high levels of baseline distress, which is perhaps unsurprising given the high prevalence of depression and anxiety in university samples (e.g. Eisenberg et al. 2007).This suggests that many of the participants, who self-selected to take part, were experiencing higher than usual symptoms of stress, anxiety or depression, and that this population may be attracted to and benefit from increased access to brief interventions such as the one described here. Follow-up data were not collected, and future research should evaluate the longevity of these effects.

A more robust test of mediation is one where the mediator is measured prior to the outcome variable (Preacher and Hayes 2008). Future studies should test mediational hypotheses (including the reverse mediation hypothesis) in longitudinal studies in order for the causal relationship between perseverative thinking, stress and symptoms to be elucidated more clearly.

The results of this study suggest that brief online mindfulness interventions, offered with or without an invitation to formal daily mindfulness meditation practice, can increase mindfulness and reduce perseverative thinking, perceived stress and symptoms of anxiety and depression in a university population. Our findings suggest that at least some people may be able to develop mindfulness skills through interventions that require no therapist resource and that these interventions may complement and extend the range of self-help materials for non-clinical populations which has to date been dominated by material based on the principles of cognitive behavioural therapy. They also suggest that education about mindfulness together with the encouragement to apply mindfulness in daily life is a helpful intervention in its own right, even in the absence of formal meditation practice. If replicated, this finding has the potential to increase accessibility and engagement in MBIs, as for some participants having to commit to regular, formal mindfulness meditation practice may be a barrier to engagement.
